# Knowledge, Attitude, Practice and Associated Factors Regarding Safe Blood Donation Among Students of Public Universities in Afghanistan: A Cross‐Sectional Study

**DOI:** 10.1002/hsr2.71757

**Published:** 2026-01-18

**Authors:** Naweedullah Noori, Bashir Ahmad Qudrati, Rohullah Sakhi

**Affiliations:** ^1^ Public Health Faculty Kabul University of Medical Sciences Kabul Afghanistan; ^2^ Environmental and Occupational Health, Public Health Faculty Kabul University of Medical Sciences Kabul Afghanistan

**Keywords:** Afghanistan, attitude, blood donation, knowledge, practice, students

## Abstract

**Background and Aims:**

Blood transfusion is a cornerstone of modern healthcare, saving millions of lives annually. However, ensuring a stable and safe blood supply remains a challenge in low‐income countries like Afghanistan. Students, as young and educated individuals, can play a vital role in promoting voluntary blood donation. This study aimed to investigate the knowledge, attitude, practice, and associated factors regarding safe blood donation among students of public universities in Afghanistan.

**Methods:**

This cross‐sectional study was conducted among 395 students between November and December 2024 using a multi‐stage stratified sampling method. Data were analyzed using SPSS 27 with frequencies, percentages, chi‐square tests, and Spearman's correlation to assess relationships between variables. Bivariate and multivariate binary logistic regression analyses evaluated associations between knowledge, attitude, practice, and demographic factors. *p*‐values < 0.05 were considered statistically significant.

**Results:**

Of the 395 distributed questionnaires, 385 were completed and returned. The response rate in this study was 97.46%. The highest number of participants in this research were in the age range of 21–25 years. The findings revealed that most students (52.7%) had adequate knowledge about safe blood donation, and the majority of students (98.4%) exhibited a favorable attitude. Only 19.2% of students reported having donated blood. The main barriers to blood donation were limited access to blood donation centers (37.4%) and not being asked to donate (59.2%). A significant association was found between faculty type and knowledge (*p *< 0.001), year of education and practice (*p *= 0.02) and marital status and practice (*p *= 0.04), based on Chi‐square tests.

**Conclusion:**

Although most students had adequate knowledge and positive attitudes regarding blood donation, their actual donation practices were low. Major barriers included logistical issues and lack of encouragement. These results underscore the need for targeted awareness campaigns, educational interventions, and improved access to blood donation facilities to enhance safe blood donation practices in Afghanistan.

## 1 Background

Blood donation is one of the important issues in healthcare, saving millions of lives each year [1]. This is in light of critical needs like trauma, surgical interventions, pregnancy‐related complications, and chronic conditions like anemia and cancer [2]. Despite its importance, supplying a stable and safe blood supply remains a global challenge, particularly in low‐income countries where the rate of voluntary blood donation is significantly lower than in high‐income countries [3]. Safe blood donation refers to the collection and transfusion of blood that is voluntary, non‐remunerated, properly screened for infections, and handled according to standard safety protocols. High‐income countries, which account for only 16% of the world's population, contribute 40% of all donations, highlighting the disparity with lower‐income regions [4, 5]. To meet basic needs, the World Health Organization (WHO) recommends that at least 1% of a nation's population be willing to donate blood. However, low‐income countries, including Afghanistan, face multiple barriers that prevent the achievement of this target [6, 7].

Afghanistan faces significant barriers to ensuring an adequate and safe blood supply, including limited infrastructure, low awareness, and cultural misconceptions [4]. While high‐income countries maintain relatively safe blood supplies, Afghanistan and similar low‐resource settings encounter substantial logistical, educational, and cultural barriers. Unsafe transfusions contribute to the spread of blood‐borne infections such as Human Immunodeficiency Virus (HIV), Treponema pallidum (causing syphilis), Hepatitis C Virus (HCV), Hepatitis B Virus (HBV), and Plasmodium species (causing malaria) [8]. An estimated 118.5 million blood donations are collected each year globally [4, 5], and the need for safe blood is growing in daily life as a result of the rise in blood‐related chronic illnesses, such as anemia, unanticipated accidents, pregnancy‐related difficulties, and various surgical operations [9]. Nevertheless, studies have also shown that blood donation rates differ based on a nation's position and resources [10]. To help patients save their lives, the WHO continues to promote voluntary and safe blood donation [6]. A study conducted in 284 blood banks in Afghanistan from 2015 to 2020 revealed that 56.93% of blood donations were family replacement‐based, while only 43.07% came from voluntary non‐remunerated donors. The prevalence of blood‐borne infections among donors was 4.36%, with Hepatitis B being the most common (2.95%) [11].

Among key population groups, university students are particularly important for promoting voluntary blood donation due to their education, awareness, and social influence. They are more likely to engage in healthy behaviors, and positive attitudes have been shown to increase the likelihood of donation. However, participation can be limited by low awareness, misconceptions, and low self‐confidence [12]. Limited research exists on blood donation in Afghanistan, with no studies specifically assessing the knowledge, attitude, and practice (KAP) and associated factors regarding safe blood donation among university students [13, 14, 15, 16]. Evidence from similar low‐resource settings such as Kenya, Syria, and Pakistan demonstrates that poor KAP levels significantly hinder voluntary blood donation and that targeted educational interventions can substantially improve donor participation [17, 18, 19]. Given Afghanistan's distinct cultural norms, limited health infrastructure, and widespread misconceptions surrounding blood donation, context‐specific KAP data are essential for designing effective and culturally appropriate interventions. Therefore, our study addresses a critical gap by providing updated KAP data from multiple major public universities in Afghanistan, which is instrumental for informing strategies to enhance voluntary donation and improve the safety of the national blood supply.

## 2 Methods

### 2.1 Study Design, Setting, and Period

This was a cross‐sectional study conducted in four major public universities in Kabul, Afghanistan: Kabul University (*n* = 9500 students), Kabul University of Medical Sciences (*n* = 1740), Kabul Polytechnic University (*n* = 3555), and Kabul Education University (*n* = 2152). These institutions represent diverse academic disciplines, including both health‐related and non‐health‐related fields. The study was carried out during November and December 2024.

### 2.2 Inclusion and Exclusion Criteria

The study included undergraduate students aged 18–30 years enrolled in the four selected public universities in Kabul who provided written informed consent. Exclusion criteria included refusal to participate, absence during data collection, or the presence of health conditions that restrict blood donation.

### 2.3 Sample Size and Sampling Method

The total number of students registered in the four major universities in 2024 was 16,549. The sample size was calculated using Cochran's formula with a 95% confidence level and a 5% margin of error, with calculations performed using Epi Info software version 7.2.6, resulting in a minimum required sample of 376 students. After accounting for a 5% non‐response rate, the final sample size was increased to 395.

Although a multi‐stage stratified sampling method was employed, the design effect was not incorporated into the sample size calculation due to logistical and resource constraints. Nevertheless, the final sample size and high response rate (97.46%) are expected to provide valid and reasonably representative results. The sampling process involved dividing the universities into four strata. Each university was then stratified by faculties, and proportional allocation was used to determine the sample size for each faculty. Within faculties, classes were randomly selected, and students were chosen randomly from those classes.

### 2.4 Data Source and Measurement

The study questionnaire, developed based on previous similar research [20, 21], aimed to assess the knowledge, attitude, and practice (KAP) of university students regarding blood donation. The questionnaire consists of 43 questions, divided into four sections, as described below: Demographic, Knowledge, Attitude, and Practice.

- Demographic Section: Five items assessing participants' age, year of education, and other relevant details.
- Knowledge Section: 16 items; 1 point for each correct answer; total score range 0–16. Categorized as Adequate knowledge (score ≥ 9) and Inadequate knowledge (score ≤ 8).
- Attitude Section: 7 items with responses scored as Agree = 3, Unsure = 2, Disagree = 1; total score 7–21. Categorized as Favorable attitude (score ≥ 13) and Unfavorable attitude (score ≤ 12).
- Practice Section: 15 items, beginning with whether the participant has ever donated blood (Yes/No). Follow‐up questions differ based on response, exploring frequency, timing, motivations, and barriers.

The questionnaire was translated into the local languages (Dari/Pashto) and back‐translated into English to ensure accuracy. A pilot study was conducted with 30 students (excluded from the main sample) to evaluate clarity, cultural appropriateness, and reliability. Minor revisions were made based on pilot feedback. The internal consistency of the questionnaire was acceptable, with Cronbach's *α* values of 0.78 for the knowledge subscale, 0.81 for the attitude subscale, and 0.74 for the practice subscale. The questionnaire was administered in person by trained research team members, who explained the study objectives and obtained written informed consent. Closed‐ended, multiple‐choice questions were designed for completion in under 10 min to minimize recall bias. Full details of the questionnaire structure, scoring procedures, and validity assessments are provided in (Supplementary Material 1).

### 2.5 Statistical Analysis

All statistical analyses were performed using IBM SPSS Statistics version 27. Descriptive statistics, including frequencies and percentages, were used to summarize categorical variables. Chi‐square tests were conducted to assess associations between categorical variables. Spearman's correlation analysis was used to evaluate the relationships between knowledge, attitude, and practice scores. Bivariate and multivariate binary logistic regression analyses were performed to identify predictors of adequate knowledge, favorable attitude, and good practice regarding blood donation. A *p*‐value of < 0.05 was considered statistically significant. All statistical tests were two‐tailed.

## 3 Results

### 3.1 Students' Sociodemographic Characteristics

Out of a total of 395 distributed questionnaires, 385 were completely filled and included in the analysis. The mean age of the participants was 21.37, with a standard deviation of 2.014. The majority of students (86.8%) were from non‐health‐related faculties. More than one‐third of the participants (37.4%) were in their first year of study. Most students were single (91.2%) and lived in their homes (62.9%). Details of participants' sociodemographic characteristics are presented in Table 1.

**Table 1:** Demographic characteristics of the participants.

Demographic characteristics		Number	Percentage
Age group	18–20	130	33.8%
	21–25	247	64.2%
	26–30	8	2.0%
Faculty type	Health‐related	51	13.2%
	Non‐health‐related	334	86.8%
Year of education	1st year	144	37.4%
	2nd year	95	24.7%
	3rd year	80	20.8%
	4th year	66	17.1%
Marital status	Single	351	91.2%
	Married	34	8.8%
Place of residence	House	242	62.9%
	Dormitory	143	37.1%

*Note:* Data are presented as frequencies and percentages to describe the sociodemographic characteristics of the participants.

### 3.2 Students' Knowledge About Safe Blood Donation

The findings of this study revealed that the majority of participants, 203 (52.7%), had adequate knowledge, while 182 (47.3%) had inadequate knowledge about safe blood donation (Table 2). According to the results of this study, 235 (61%) of the participants knew their blood group, whereas 150 (39%) did not. Most participants, 205 (53.2%), indicated that smokers and alcoholics cannot donate blood, 117 (30.4%) believed they can, and 63 (16.4%) were unsure. Additionally, 240 (62.3%) of participants recognized that HIV can be transmitted through blood, 56 (14.5%) believed it cannot, and 89 (23.1%) were unsure.

**Table 2:** Students' knowledge about safe blood donations.

Variables	Yes		No		Do not know	
	*N*	%	*N*	%	*N*	%
1. Do you know your blood group?	235	61%	150	39%		
2. Blood volume recovery within 24–48 h	93	24.2%	135	35.1%	157	40.8%
3. Blood donation by women during menstruation	20	5.2%	263	68.3%	102	26.5%
4. Can pregnant women donate blood?	25	6.5%	297	77.5%	63	16.4%
5. Can breastfeeding women donate blood?	85	22.1%	227	59%	73	19%
6. Can you donate blood if you feel sick or have a fever?	67	17.4%	224	58.2%	94	24.4%
7. Can you donate blood if you have had a tattoo or acupuncture in the past 6 months?	101	26.2%	140	36.4%	144	37.4%
8. Do people need to pay money to receive blood when they need it?	111	28.8%	238	61.8%	36	9.4%
9. Can a person become ill after donating blood?	104	27%	230	59.7%	51	13.2%
10. Can a person infected with hepatitis B or C donate blood?	19	4.9%	306	79.5%	60	15.6%
11. Can individuals under the age of 18 donate blood?	151	39.2%	151	39.2%	83	21.6%
12. Is your blood tested before being transfused to other people?	296	76.9%	50	13%	39	10.1%
13. Can smokers and alcoholics donate blood?	117	30.4%	205	53.2%	63	16.4%
14. Can someone donate blood if they haven′t had enough sleep?	208	54%	87	22.6%	90	23.4%
15. Is HIV transferrable through blood?	240	62.3%	56	14.5%	89	23.1%
16. Is hepatitis transferrable through blood?	190	49.4%	67	17.4%	128	33.2%

*Note:* Data are presented as frequencies and percentages to describe students' knowledge about safe blood donation.

### 3.3 Students' Attitudes About Safe Blood Donation

The findings of this study revealed that the majority of participants, 379 (98.4%), had a favorable attitude, while only 6 (1.6%) had an unfavorable attitude toward safe blood donation (Table 3). According to the results of this study, most participants, 357 (92.7%), considered blood donation an important and humane duty, while 20 (5.2%) were unsure, and 8 (2.1%) did not consider blood donation as an important and humane duty. In total, 277 (71.9%) of participants reported willingness to donate blood if requested, 23 (6%) were unwilling, and 85 (22.1%) were unsure. Regarding misconceptions, 148 (38.4%) of participants were unsure whether donating blood causes anemia, weakness, or weight loss; 127 (33%) believed it does, and 110 (28.6%) disagreed.

**Table 3:** Students' attitude about safe blood donations.

Variables	Agree		Unsure		Disagree	
	*N*	%	*N*	%	*N*	%
1. Blood donation is an important act and a human duty	357	92.7%	20	5.2%	8	2.1%
2. I will donate blood if I am asked to do so	277	71.9%	85	22.1%	23	6.0%
3. Blood donation helps patients in need	363	94.3%	14	3.6%	8	2.1%
4. I encourage people to donate blood	306	79.5%	61	15.8%	18	4.7%
5. Everyone should have awareness about blood donation	366	95.1%	14	3.6%	5	1.3%
6. I think I do not have enough blood to donate	151	39.2%	173	45%	61	15.8%
7. Blood donation causes anemia, weakness, and weight loss	127	33.0%	148	38.4%	110	28.6%

*Note:* Data are presented as frequencies and percentages to describe students' attitudes toward safe blood donation.

### 3.4 Students' Practices About Safe Blood Donation

The findings of this study showed that the majority of participants, 311 (80.8%), had never donated blood, while 74 (19.2%) of the participants reported that they had donated blood (Table 4). According to the results of this study, among all participants, 56 (75.7%) had donated blood once, 14 (18.9%) had donated blood 2–5 times, and only 4 (5.4%) had donated blood more than five times. Among participants, 30 (40.5%) had donated blood 1 year ago, 25 (33.8%) more than 1 year ago, and 19 (25.7%) within the past 1–6 months. Among motivations, 64 (16.6%) donated blood to support patients in need, 52 (13.5%) for friends or relatives, and 9 (2.3%) in exchange for money or gifts (Table 4). The barriers preventing these individuals from donating blood were reported as follows: 43 (11.2%) reported that they could not donate blood due to sickness, 144 (37.4%) reported that a lack of access to blood donation centers prevented them from donating, and 228 (59.2%) reported that no one had ever asked them to donate blood.

**Table 4:** Students' practice about safe blood donation.

A. Donated blood (*n* = 74, 19.2%)		
A1. Donation frequency and timing (among donors, *n* = 74)		
Variable	Category	*N* (%)
Number of blood donations	Once	56 (75.7%)
	2–5 times	14 (18.9%)
	More than five times	4 (5.4%)
Time since last donation	1–6 Months	19 (25.7%)
	1 year ago	30 (40.5%)
	More than one year ago	25 (33.8%)

**A2. Motivations for blood donation (multiple responses allowed; among donors, *n*** = **74)**		
Motivation	Yes, *n* (%)	No, *n* (%)
Support patients in need	64 (16.6%)	10 (2.6%)
Health maintenance	42 (10.9%)	32 (8.3%)
Free health evaluation	50 (13%)	24 (6.2%)
Purify or stimulate blood production	34 (8.8%)	40 (10.4%)
For relatives/friends	52 (13.5%)	22 (5.7%)
For money/gifts	9 (2.3%)	65 (16.9%)

B. Not donated blood (*n* = 311, 80.8%)		
B1. Barriers to blood donation (multiple responses allowed; among non‐donors, *n* = 311)		
Barriers	Yes, *n* (%)	No, *n* (%)
Because I am sick	43 (11.2%)	268 (69.6%)
Limited access to donation centers/unfamiliarity	144 (37.4%)	167 (43.4%)
Fear of contracting diseases	76 (19.7%)	235 (61%)
Fear of needles/blood	68 (17.7%)	243 (63.1%)
No one asked me	228 (59.2%)	83 (21.6%)
Family disapproval	80 (20.8%)	231 (60%)

*Note:* Data are presented as frequencies and percentages to describe students' practices regarding safe blood donation. Percentages for donation frequency and time since last donation were calculated among donors only (*n* = 74). Percentages in the motivation and barrier items were calculated based on the total in each group (donors = 74, non‐donors = 311). Multiple responses were permitted; therefore, percentages may exceed 100%.

#### 3.4.1 Association of Students' Sociodemographic Characteristics With Knowledge, Attitude, and Practices Regarding Safe Blood Donation

Although students aged 21–25 years appeared to have slightly better knowledge and attitudes, statistical analysis using the Chi‐square test revealed no significant differences in knowledge (*χ*² = 1.194, *p* = 0.55) or attitude (*χ*² = 0.133, *p* = 0.94) across age groups. For the age group 26–30 years, where some expected cell counts were less than 5, Fisher‐Freeman‐Halton Exact Test was applied (Knowledge: *p* = 0.546, Attitude: *p* = 1.000, Practice: *p* = 0.225), and results were consistent with the Chi‐square analysis. Similarly, blood donation practices did not differ significantly by age (χ² = 2.675, *p* = 0.26). However, a significant association was observed between faculty type and knowledge (χ² = 11.191, *p* < 0.001), indicating that students from health‐related faculties had higher knowledge levels compared to those from non‐health‐related faculties. No significant association was found between faculty type and attitude (χ² = 0.062, *p* = 0.80) or practice (χ² = 1.488, *p* = 0.22). However, a significant association was observed between academic year and practice (χ² = 10.337, *p* = 0.02), indicating that students in higher academic years were more likely to have donated blood. Additionally, marital status showed a significant association with practice (χ² = 4.142, *p* = 0.04), with married students reporting a higher rate of donation (Table 5).

**Table 5:** Association between demographic characteristics and Knowledge, attitude, and practice.

Variables	Knowledge level				Attitude level				Practice level			
	Adequate	Inadequate	Chi‐square	*p* value	Favorable	Unfavorable	Chi‐square	*p* value	Donated	Not donated	Chi‐square	*p* value
**Age group**												
18–20	72 (55.4%)	58 (44.6%)	1.194	0.55	128 (98.5%)	2 (1.5%)	0.133	0.94	21 (16.2%)	109 (83.8%)	2.675	0.26
21–25	128 (51.8%)	119 (48.2%)			243 (98.4%)	4 (1.6%)			50 (20.2%)	197 (79.8%)		
26–30	3 (37.5%)	5 (62.5%)			8 (100%)	0			3 (37.5%)	5 (62.5%)		
**Faculty type**												
Health‐related	38 (74.5%)	13 (25.5%)	11.191	< 0.001	50 (98%)	1 (2%)	0.062	0.80	13 (25.5%)	38 (74.5%)	1.488	0.22
Non‐health‐related	165 (49.4%)	169 (50.6%)			329 (98.5%)	5 (1.5%)			61 (18.3%)	273 (81.7%)		
**Year of education**												
1st year	75 (52.1%)	69 (47.9%)	0.504	0.92	144 (100%)	0	4.327	0.23	16 (11.1%)	128 (88.9%)	10.337	0.02
2nd year	52 (54.7%)	43 (45.3%)			92 (96.8%)	3 (3.2%)			23 (24.2%)	72 (75.8%)		
3rd year	40 (50%)	40 (50%)			78 (97.5%)	2 (2.5%)			21 (26.3%)	59 (73.7%)		
4th year	36 (54.5%)	30 (45.5%)			65 (98.5%)	1 (1.5%)			14 (21.2%)	52 (78.8%)		
**Marital status**												
Single	187 (53.3%)	164 (46.7%)	0.481	0.49	346 (98.6%)	5 (1.4%)	0.465	0.50	63 (17.9%)	288 (82.1%)	4.142	0.04
Married	16 (47.1%)	18 (52.9%)			33 (97.1%)	1 (2.9%)			11 (32.4%)	23 (67.6%)		
**Place of residence**												
House	131 (54.1%)	111 (45.9%)	0.516	0.47	239 (98.8%)	3 (1.2%)	0.432	0.51	44 (18.2%)	198 (81.8%)	0.453	0.50
Dormitory	72 (50.3%)	71 (49.7%)			140 (97.9%)	3 (2.1%)			30 (21%)	113 (79%)		

*Note:* All analyses were primarily performed using the Chi‐square test and cross‐tabulation. For cells with expected counts less than 5 (particularly in the 26–30 age group), Fisher‐Freeman‐Halton Exact Test was applied. Exact *p*‐values are reported where applicable (Knowledge: *p* = 0.546, Attitude: *p* = 1.000, Practice: *p* = 0.225). Statistical significance was considered at *p* < 0.05.

The Spearman correlation analysis revealed a weak positive correlation between attitude and practice (ρ = 0.114, *p* = 0.025) and a weak negative correlation between knowledge and practice (ρ = –0.101, *p* = 0.048), both statistically significant. However, the correlation between knowledge and attitude was very weak and not statistically significant (ρ = 0.039, *p* = 0.44) (Table 6).

**Table 6:** Correlation between knowledge, attitude, and practice scores.

Variable	Mean ± SD (1st Variable)	Mean ± SD (2nd Variable)	Spearman's ρ	*p* value
Attitude‐practice	18.38 ± 2.15	1.81 ± 0.40	0.114	0.025
Knowledge‐practice	8.59 ± 2.67	1.81 ± 0.40	−0.101	0.048
Knowledge‐attitude	8.59 ± 2.67	18.38 ± 2.15	0.039	0.44

*Note:* Correlation between continuous scores was assessed using Spearman's rank correlation coefficient (ρ). *p* < 0.05 was considered statistically significant.

Logistic regression analysis (Table 7) further examined the influence of sociodemographic variables on KAP outcomes. After adjusting for potential confounders, most demographic factors, including age group, marital status, and place of residence, were not significantly associated with knowledge, attitude, or practice in the multivariate model. However, year of education was significantly associated with practice (AOR = 0.73, *p* = 0.02), and faculty type showed a borderline significant association with practice (AOR = 2.14, *p* = 0.05). Faculty type was significantly associated with knowledge in both bivariate analysis (COR = 0.33, *p* < 0.001) and multivariate analyses (AOR = 0.29, *p* = 0.001). For instance, while the bivariate analysis indicated that married students were more likely to have donated blood (COR = 0.46, 95% CI: 0.21–0.99, *p* = 0.04), this association was no longer significant in the adjusted model (AOR = 0.52, 95% CI: 0.23–1.13, *p* = 0.10). These findings suggest that although some demographic factors such as academic year and marital status are associated with students' blood donation behavior in bivariate analyses, year of education and faculty type have independent predictive effects on knowledge and practice in the multivariate model, which should be acknowledged when interpreting the results.

**Table 7:** Bivariate and multivariate logistic regression analysis of sociodemographic predictors of knowledge, attitude and practice regarding safe blood donation.

Demographic characteristics	Knowledge		Attitude		Practice	
	COR (95% CI, *p* value)	AOR (95% CI, *p*‐value)	COR (95% CI, *p* value)	AOR (95% CI, *p* value)	COR (95% CI, *p* value)	AOR (95% CI, *p* value)
Age group	NA (*p* = 0.55)	0.81 (0.51–1.29, *p* = 0.38)	NA (*p* = 0.94)	1.97 (0.30–13.08, *p* = 0.48)	NA (*p* = 0.26)	0.91 (0.50–1.65, *p* = 0.75)
Faculty type	0.33 (0.17–0.65, *p* = 0.001)	0.29 (0.14–0.58, *p* = 0.001)	1.32 (0.15–11.50, *p* = 0.80)	1.98 (0.17–22.72, *p* = 0.58)	1.53 (0.77–3.05, *p* = 0.22)	2.14 (1.00–4.60, *p* = 0.05)
Year of education	NA (*p* = 0.92)	1.17 (0.94–1.45, *p* = 0.16)	NA (*p* = 0.23)	0.54 (0.22–1.31, *p* = 0.17)	NA (*p* = 0.02)	0.73 (0.55–0.96, *p* = 0.02)
Marital status	0.78 (0.39–1.58, *p* = 0.49)	0.76 (0.37–1.58, *p* = 0.46)	0.48 (0.05–4.20, *p* = 0.50)	0.55 (0.06–5.10, *p* = 0.60)	0.46 (0.21–0.99, *p* = 0.04)	0.53 (0.24–1.17, *p* = 0.12)
Place of residence	0.86 (0.57–1.30, *p* = 0.47)	0.79 (0.52–1.22, *p* = 0.29)	0.59 (0.12–2.94, *p* = 0.51)	0.58 (0.11–2.98, *p* = 0.51)	0.84 (0.50–1.41, *p* = 0.50)	0.83 (0.49–1.43, *p* = 0.51)

*Note:* Bivariate and multivariate logistic regression analyses were conducted to determine the associations between sociodemographic factors and Knowledge, Attitude, and Practice. Pp < .050.05 was considered statistically significant.

Abbreviations: AOR = Adjusted Odds Ratio, CI = Confidence Interval, COR = Crude Odds Ratio.

## 4 Discussion

This cross‐sectional study assessed the knowledge, attitude, practice, and associated factors regarding safe blood donation among students of public universities in Afghanistan. Only 19.2% of participants had ever donated blood, despite the majority demonstrating favorable attitudes (98.4%) and adequate knowledge (52.7%). Key barriers included a lack of requests to donate, limited access to donation centers, and fear‐related concerns. While academic year and marital status were associated with donation behavior in bivariate analysis, they were not independent predictors in multivariate analysis.

The findings of this study showed that the majority of students (20.2%) who donated blood were aged 21–25 years. This finding is lower than that reported in a study [11] conducted in 284 blood donation centers across 34 provinces of Afghanistan (42.92%). Moreover, this result does not align with studies conducted in Kenya (42.24%) [17], Tanzania (44.7%) [22], and Pakistan (45.6%) [19]. These discrepancies likely reflect contextual differences across countries, including variations in health literacy, cultural norms surrounding blood donation, the maturity of voluntary versus replacement‐based donation systems, and the strength of national awareness campaigns. Differences in students' access to donation opportunities and exposure to health information may further contribute to these variations.

The percentage of students with adequate knowledge regarding safe blood donation was 52.7%. No significant differences were observed between the level of knowledge and demographic variables. This result is slightly lower than that reported in a study conducted in Gaza (54.7%) [23] and lower than studies in Saudi Arabia (71.1%) [24], Nigeria (81.6%) [25], Iran (98%) [26], and Syria (99.7%) [18]. These differences may stem from variations in access to information and the availability of blood donation centers. High‐income countries benefit from widespread educational resources, boosting public awareness, while low‐income countries like Afghanistan face limited resources, leading to lower general knowledge.

Although students from health‐related faculties demonstrated significantly higher knowledge levels, as reflected in the results section, this difference did not extend to attitude or practice, which showed no significant association with faculty type. This pattern is frequently reported in KAP studies, where academic exposure improves theoretical understanding but does not necessarily influence behavioral intentions or actual practices. Attitude and practice are often influenced by broader cultural norms, personal beliefs, previous donation experiences, and the availability of accessible donation opportunities rather than academic knowledge alone.

The findings of this study showed that 61% of students were aware of their blood group. This result is similar to a study conducted in Gaza (66.9%) [23] but lower than studies in Ethiopia (50.66%) [12], Northern India (95.7%) [27], and Nigeria (96%) [28]. Differences may reflect variations in educational systems and emphasis on health awareness across countries. In advanced systems, students often learn about health topics, including their blood type, during school, while in less developed settings, such information is less accessible.

The results of this study showed that the majority of students (98.4%) had a favorable attitude toward blood donation. This finding is higher than that reported in a study conducted in Gaza, where 73.3% of participants reported having a positive attitude [23]. This difference may be influenced by cultural and social factors. In some communities, negative attitudes toward blood donation arise from misconceptions, fear of side effects, or distrust in healthcare systems, while communities with effective awareness programs tend to have more positive attitudes.

Notably, only 14.5% of the students had donated blood once. This finding is slightly lower than the study conducted in Ethiopia (19.3%) [12] and lower than studies in Malaysia (29.7%) [29], Nigeria (35.4%) [30], India (47.5%) [31], and Namibia (28%) [32]. Such differences may result from variations in access to blood donation centers or the availability of incentives. In Afghanistan, national programs to promote donation may be limited in scale compared to other countries with well‐organized initiatives.

Our results also showed that 13.5% of students donated blood to relatives and friends. This finding is slightly similar to a study in Northern India (14.8%) [27] but lower than a previous study conducted in Afghanistan (56.93%) [11]. This discrepancy may be related to cultural and social factors. In Afghanistan, blood donation may primarily occur within families and for close relatives, whereas in other countries, blood donation is more commonly done for public health purposes and medical facilities.

The reasons for not donating blood among students included lack of access to blood donation centers (37.4%), fear of infection (19.7%), and fear of needles (17.7%). These findings are generally consistent with studies conducted in Saudi Arabia [33] and Sub‐Saharan Africa [34]. This highlights the impact of psychological factors (e.g., fear of needles or infection) and infrastructural limitations (e.g., insufficient blood donation centers) on reducing donation rates.

This study provides valuable insights into the knowledge, attitude, practice, and associated factors regarding blood donation among university students in Afghanistan, a context with limited prior research. Given the scarcity of voluntary donation programs, limited health data systems, and restricted access to research populations in Afghanistan, generating empirical KAP data from four major public universities provides contextually important evidence. While the study is not pioneering, it incrementally contributes to understanding trends and determinants of blood donation in this population.

The study was conducted in four major public universities, which may limit the generalizability of the findings. Cultural barriers may have restricted participation among female students, and their perspectives might differ from those of male participants. Additionally, the design effect was not applied in the sample size calculation due to resource constraints. Nevertheless, careful stratified sampling was used to maintain representativeness and provide meaningful insights. The study did not assess whether participants had received any prior formal or informal education regarding blood donation, which may have influenced their knowledge scores. More detailed discipline‐ or department‐specific analyses were not conducted and could be explored in future studies.

## 5 Conclusion

Despite adequate knowledge and highly favorable attitudes among university students in Afghanistan, actual blood donation rates remain low. Integrating blood donation education into university curricula and organizing regular on‐campus blood drives could help overcome barriers such as limited access and insufficient encouragement, enhancing voluntary donation rates in this population.

## Author Contributions

**Naweedullah Noori:** writing – original draft, methodology, conceptualization, investigation, data collection, visualization. **Bashir Ahmad Qudrati:** writing – original draft, methodology, writing – review and editing, software, validation, conceptualization, investigation, formal analysis, project administration, data curation, supervision. **Rohullah Sakhi:** supervision, validation, conceptualization.

## Funding

The authors received no specific funding for this work.

## Ethics Statement

This study received ethical approval from the Institutional Review Board (IRB) of the Faculty of Public Health, Kabul University of Medical Sciences. The protocols of the current study are in compliance with the ethical considerations of the institutional review board and with the principles of the Declaration of Helsinki.

## Conflicts of Interest

The authors declare no conflicts of interest.

## Transparency Statement

The lead author, Dr. Bashir Ahmad Qudrati, affirms that this manuscript is an honest, accurate, and transparent account of the study being reported; that no important aspects of the study have been omitted; and that any discrepancies from the study as planned have been explained.

## Supporting information

Supplementary Material.

## Data Availability

Data cannot be shared publicly for reasons of ethical restriction and anonymity. Data are available upon request from Dr. Bashir Ahmad Qudrati, Kabul University of Medical Sciences, Kabul, Afghanistan, via email: bashirqudrati786@gmail. com.
