# Supplementary material for: Knowledge, Attitude, Practice and Associated Factors Regarding Safe Blood Donation Among Students of Public Universities in Afghanistan: A Cross‐Sectional Study
Source: Health Sci Rep. 2026 Jan 18;9(1):e71757. doi: 10.1002/hsr2.71757 (PMC12813413; doi:10.1002/hsr2.71757)
Supplement: Supplementary file 1 — Supplementary Material. [file HSR2-9-e71757-s001.docx]

**Supplementary Material 1: Study Questionnaire and Measurement Details**

**Introduction**

This supplementary file provides detailed information on the structure, development, scoring, validity, and reliability of the questionnaire used in this study. The instrument was developed based on previously validated KAP tools from similar research (References 20 and 21). The questionnaire consisted of 43 items divided into four major sections and was designed to assess the knowledge, attitudes, and practices (KAP) of university students regarding blood donation. The overall aim was to ensure conceptual clarity, cultural appropriateness, internal consistency, and ease of completion within a short time frame (approximately 8–10 minutes).

**Questionnaire Structure and Scoring**

The questionnaire comprised four sections: Demographics, Knowledge, Attitude, and Practice.

1. Demographic Section:

This section included 5 items questions related to participants’ age, year of education, and other relevant personal and academic information.

1. Knowledge Section:

This section contained 16 questions designed to assess participants’ understanding of blood donation. Each correct answer was awarded 1 point, and incorrect answers received 0 points.

- - Total score range: 0–16
  - Knowledge levels were categorized as:
    - Adequate knowledge: score ≥ 9
    - Inadequate knowledge: score ≤ 8

Internal consistency: Cronbach’s α = 0.78

1. Attitude Section:

This section included 7 items to assess participants’ attitudes toward blood donation. Each item had three response options:

- - Agree = 3 points
  - Unsure = 2 points
  - Disagree = 1 point
  - Total score range: 7–21
  - Attitude levels were categorized as:
    - Favorable attitude: score ≥ 13
    - Unfavorable attitude: score ≤ 12

Internal consistency: Cronbach’s α = 0.81

1. Practice Section:

This section included 15 questions that evaluated participants’ blood donation behaviors.
It began with a screening question — “Have you ever donated blood?” (Donated/Not Donated).

- - If the response was “Donated” the following questions explored the frequency of donation, time since last donation, and motivations for donating.
  - If the response was “Not Donated” subsequent questions examined the reasons for not donating and barriers preventing participation in blood donation activities.

Internal consistency: Cronbach’s α = 0.74

**Sampling and Data Collection Procedures**

Universities were stratified by faculty type, and proportional allocation was used to determine the sample size for each faculty. Classes were randomly selected, and students were chosen using simple random techniques.

Data collection was conducted in person by trained research team members. The team:

- explained the study objectives,
- obtained written informed consent,
- assured confidentiality and anonymity, and
- supervised on-site completion to avoid peer influence and information sharing.

**Translation, Validity, and Pilot Testing**

The original questionnaire (in English) was translated into Dari and Pashto to ensure clarity and comprehension.

A professional bilingual expert performed a back-translation into English. The research team compared the back-translated version with the original to ensure semantic, conceptual, and cultural equivalence. Minor adjustments were made to resolve inconsistencies.

Content validity was assessed by a panel of five experts in public health, hematology, and health education.

Experts evaluated each item for:

- relevance,
- clarity,
- cultural acceptability,
- and alignment with study objectives.

Items with a Content Validity Index (CVI) below 0.80 were revised for clarity.

A pilot study was conducted with 30 undergraduate students (excluded from the main sample). The pilot assessed:

- clarity of items,
- logical flow,
- comprehension time,
- and cultural appropriateness.

Minor revisions included:

- simplifying terminology,
- improving the clarity of knowledge items,
- reorganizing response options in the practice section,
- removing ambiguous phrasing.

The pilot confirmed that participants completed the questionnaire within 8–10 minutes, with minimal confusion.

**English Version of the Questionnaire**

**Section A: Socio demography**

**Instruction**: Circle or check the box that corresponds to the best answer.

**Age**

**Faculty Type**

**☐** Health-related

☐ Non-health-related

**Year of Education**

**☐** 1st year students

**☐** 2nd year students

**☐** 3rd year students

**☐** 4th year students

**Marital status**

**☐** Single

☐ Married

**Place of residence**

**☐** House

**☐** Dormitory

**Section B: Knowledge About Safe Blood Donation**

| **Variables** | **Yes** | **No** | **Do not Know** |
| --- | --- | --- | --- |
| Do you know your blood group? | ☐ | ☐ | ☐ |
| Blood volume recovery within 24–48 hours | ☐ | ☐ | ☐ |
| Blood donation by women during menstruation | ☐ | ☐ | ☐ |
| Can pregnant women donate blood? | ☐ | ☐ | ☐ |
| Can breastfeeding women donate blood? | ☐ | ☐ | ☐ |
| Can you donate blood if you feel sick or have a fever? | ☐ | ☐ | ☐ |
| Can you donate blood if you have had a tattoo or acupuncture in the past 6 months? | ☐ | ☐ | ☐ |
| Do people need to pay money to receive blood when they need it? | ☐ | ☐ | ☐ |
| Can a person become ill after donating blood? | ☐ | ☐ | ☐ |
| Can a person infected with hepatitis B or C donate blood? | ☐ | ☐ | ☐ |
| Can individuals under the age of 18 donate blood? | ☐ | ☐ | ☐ |
| Is your blood tested before being transfused to other people? | ☐ | ☐ | ☐ |
| Can smokers and alcoholics donate blood? | ☐ | ☐ | ☐ |
| Can someone donate blood if they haven’t had enough sleep? | ☐ | ☐ | ☐ |
| Is HIV transferrable through blood? | ☐ | ☐ | ☐ |
| Is hepatitis transferrable through blood? | ☐ | ☐ | ☐ |

**Section C: Attitude About Safe Blood Donation.**

| **Variables** | **Agree** | **Unsure** | **Disagree** |
| --- | --- | --- | --- |
| 1.Blood donation is an important act and a human duty | ☐ | ☐ | ☐ |
| 2. I will donate blood if I am asked to do so | ☐ | ☐ | ☐ |
| 3. Blood donation helps patients in need | ☐ | ☐ | ☐ |
| 4. I encourage people to donate blood | ☐ | ☐ | ☐ |
| 5. Everyone should have awareness about blood donation | ☐ | ☐ | ☐ |
| 6. I think I do not have enough blood to donate | ☐ | ☐ | ☐ |
| 7. Blood donation causes anemia, weakness, and weight loss | ☐ | ☐ | ☐ |

**Section D: Practice About Safe Blood Donation.**

| **Have you ever donated blood?** | | | |
| --- | --- | --- | --- |
| **Donated Blood** | | | |
| **Variables** | | | |
| Number of blood donations | ☐ Once | ☐ 2-5 times | ☐ More than 5 times |
| **Variables** | | | |
| Time since last donation | ☐ 1-6 Month | ☐ 1 year ago | ☐ More than years ago |
| **Motivations** | | | |
| I have donated blood to support patients in need | | ☐ Yes | ☐ No |
| Donation for health maintenance | | ☐ Yes | ☐ No |
| I have donated blood for a free health evaluation | | ☐ Yes | ☐ No |
| I have donated blood to purify and stimulate blood production | | ☐ Yes | ☐ No |
| I have donated blood for relatives and friends who need it | | ☐ Yes | ☐ No |
| I have donated blood to receive money and gifts | | ☐ Yes | ☐ No |
| **Not Donated Blood** | | | |
| **Barriers** | | | |
| Because I am sick | | ☐ Yes | ☐ No |
| Limited access to donation centers or unfamiliarity with procedures | | ☐ Yes | ☐ No |
| Because of the fear of contracting diseases | | ☐ Yes | ☐ No |
| Fear of needles or blood | | ☐ Yes | ☐ No |
| Because no one has asked me | | ☐ Yes | ☐ No |
| Family disapproval | | ☐ Yes | ☐ No |
